# Halophilic Bacteria as a Source of Novel Hydrolytic Enzymes

**DOI:** 10.3390/life3010038

**Published:** 2013-01-10

**Authors:** María de Lourdes Moreno, Dolores Pérez, María Teresa García, Encarnación Mellado

**Affiliations:** Department of Microbiology and Parasitology, University of Seville, nº2. 41012, Sevilla, Spain; E-Mails: lmoreno@us.es (M.L.M.); lpg@us.es (D.P.); mtgg@us.es (M.T.G.)

**Keywords:** halophiles, extremophiles, hydrolases, saline environments

## Abstract

Hydrolases constitute a class of enzymes widely distributed in nature from bacteria to higher eukaryotes. The halotolerance of many enzymes derived from halophilic bacteria can be exploited wherever enzymatic transformations are required to function under physical and chemical conditions, such as in the presence of organic solvents and extremes in temperature and salt content. In recent years, different screening programs have been performed in saline habitats in order to isolate and characterize novel enzymatic activities with different properties to those of conventional enzymes. Several halophilic hydrolases have been described, including amylases, lipases and proteases, and then used for biotechnological applications. Moreover, the discovery of biopolymer-degrading enzymes offers a new solution for the treatment of oilfield waste, where high temperature and salinity are typically found, while providing valuable information about heterotrophic processes in saline environments. In this work, we describe the results obtained in different screening programs specially focused on the diversity of halophiles showing hydrolytic activities in saline and hypersaline habitats, including the description of enzymes with special biochemical properties. The intracellular lipolytic enzyme LipBL, produced by the moderately halophilic bacterium *Marinobacter lipolyticus,* showed advantages over other lipases, being an enzyme active over a wide range of pH values and temperatures. The immobilized LipBL derivatives obtained and tested in regio- and enantioselective reactions, showed an excellent behavior in the production of free polyunsaturated fatty acids (PUFAs). On the other hand, the extremely halophilic bacterium, *Salicola marasensis* sp. IC10 showing lipase and protease activities, was studied for its ability to produce promising enzymes in terms of its resistance to temperature and salinity.

## 1. Introduction

Microbial life can be found over a wide range of extreme conditions (salinity, pH, temperature, pressure, light intensity, oxygen and nutrient conditions). Hypersaline environments constitute typical examples of environments with extreme conditions due to their high salinity, exposure to high and low temperatures, low oxygen conditions and in some cases, high pH values. Bacteria and Archaea are the most widely distributed organisms in these environments [1].

The classification of Kushner and Kamekura [2] defines different categories of halophilic microorganisms based on the optimal salt concentration wherein they show optimal growth, and it includes four categories: non-halophilic organisms are defined as those requiring less than 1% NaCl, whereas if they can tolerate high salt concentrations are considered as halotolerant microorganisms. With respect to halophilic microorganisms, the classification distinguishes among slight halophiles (marine bacteria), which grow best in media with 1% to 3% NaCl, moderate halophiles, growing best in media with 3% to 15% NaCl, and extreme halophiles, which show optimal growth in media containing 15% to 30% NaCl.

Halophiles have developed two different adaptive strategies to cope with the osmotic pressure induced by the high NaCl concentration of the normal environments they inhabit [3,4]. The halobacteria and some extremely halophilic bacteria accumulate inorganic ions in the cytoplasm (K^+^, Na^+^, Cl^−^) to balance the osmotic pressure of the medium, and they have developed specific proteins that are stable and active in the presence of salts. In contrast, moderate halophiles accumulate in the cytoplasm high amounts of specific organic osmolytes, which function as osmoprotectants, providing osmotic balance without interfering with the normal metabolism of the cell [5].

In recent years, halophilic microorganisms have been explored for their biotechnological potential in different fields [6]. The applications range from the use of different products, such as the compatible solutes, biopolymers or carotenoids in a variety of industries or the use of these microorganisms in environmental bioremediation processes. Besides being intrinsically stable and active at high salt concentrations, halophilic enzymes offer important opportunities in biotechnological applications, such as food processing, environmental bioremediation and biosynthetic processes. In this sense, the finding of novel enzymes showing optimal activities at various ranges of salt concentrations, temperatures and pH values is of great importance [7]. It is important to highlight that the use of enzymes from halophiles in industrial applications are not limited to their stability at high salt concentrations, since these extremozymes usually are also tolerant to high temperatures and they are stable in presence of organic solvents [8].

In general, low water activity (a_w_) produces conformation changes in the enzymes affecting the catalytic activity due to the reduced hydration. However, halophilic enzymes are active and stable in media with low water activity because even at low a_w_ enough water is present to retain suitable charge distribution at the active site maintaining the conformation of the enzyme [9]. Organic solvents with a log P lower than 2, generally are considered to cause enzyme denaturation, producing the distortion of the water-biocatalyst interactions. However, several studies have been reported on hydrolases produced by extremophiles isolated from saline environments that are very stable in solutions containing organic solvents [10].

This review will be focused on the diversity and biotechnological applications of the novel described enzymes from two groups of bacteria: the moderate and the extreme halophiles. The adaptation to live in hypersaline environments give rise to these extremophiles advantages to be exploited from a biotechnological point of view.

## 2. Diversity of Halophilic Bacteria Showing Hydrolytic Activities

Most research studies performed on hypersaline environments have been focused on the microbial diversity and ecology of these environments and progress in understanding the systematic, cellular function and metabolic activities of halophiles have been achieved. However, environmental studies based on the diversity of halophiles showing hydrolytic activities in saline habitats remain largely unexplored; however, the hydrolysis of high-molecular-weight biopolymers constitutes an initial step in the metabolism of organic compounds in the different ecosystems, playing an important role in the geochemical cycling of nutrients in salterns. In this sense, these studies must be considered of great interest due to the great biotechnological potential exhibited by these enzymes. 

In the last few years, different screening programs have been carried out to study the diversity of microorganisms producing hydrolytic enzymes throughout direct plating on agar media supplemented with specific substrates for the enzymes of interest. A limited number of halophilic bacteria showing hydrolytic activities have been isolated and characterized from different hypersaline habitats, such as solar salterns, salt lakes, saline deserts and saline deposits (Table 1).

### 2.1. Screening on Solar Salterns

Sánchez-Porro *et al.* [11] showed the abundance of five hydrolases including amylase, protease, lipase, DNase and pullulanase in a community of moderate halophiles isolated from water and sediment (superficial layer) in Spanish salterns, describing amylase producers as the most abundant isolates. Most environmental isolates able to produce hydrolytic enzymes belonged to the Gram-negative genera *Salinivibrio* and *Halomonas*, two genera widely distributed in hypersaline environments [12,13]. Among the Gram-positive, representatives of the genera *Bacillus* and *Salibacillus* were predominant. Isolates producing lipases were very diverse from a phylogenetic point of view. However, the pullulanase producers were limited to representatives of the genera *Salinivibrio*, *Halomonas* and *Bacillus-Salibacillus*. Four strains presented the five hydrolytic activities tested and multiple hydrolytic activities were detected in a few strains. A moderately halophilic bacterium (strain SM19^T^) displaying lipolytic activity was isolated and characterized. Strain SM19^T^ is a Gram-negative rod that grows optimally in culture media containing 7.5% NaCl under aerobic conditions and was classified in the genus *Marinobacter* as a novel species *Marinobacter lipolyticus* sp. nov. [14].

Moreno *et al.* [15] studied the diversity of extreme halophiles producing lipase, protease, amylase and nuclease in crystallizer ponds at two solar salterns in South Spain, concluding that 70% of total of the hydrolytic isolates were also amylase producers and no DNAse producers were detected among the screened population. Multiple hydrolytic activities were also found in this study. A clear dominance of Archaea was found, although a population of Bacteria was also present, accounting for around 7% of the total hydrolytic community isolated. Only three isolated strains were characterized as extremely halophilic bacteria (genera *Salicola*, *Salinibacter* and *Pseudomonas*). The genera *Salinibacter* and *Salicola* have been previously reported to compete with halophilic members of the Archaea in crystallizer ponds of different solar salterns [16,17]. The extremely halophilic strain, *Salicola* strain IC10 was selected for further studies for two main reasons, the potential biotechnological applications due to its dual hydrolytic activity (lipase and protease) and its use as a model for fundamental studies aimed to unravel the adaptations of halophilic enzymes, which allow them to be stable and active at high salt concentrations.

**Table 1:** Microorganisms able to produce hydrolytic enzymes isolated from different hypersaline environments.

Isolation Site	Hydrolytic Activity Assayed	Most Abundant Hydrolytic Activity	Isolate Affiliation	References
Salterns in Almeria, Cadiz and Huelva (Spain)	amylase protease lipase DNase pullulanase	amylase	*Salinivibrio* *Halomonas* *Chromohalobacter* *Bacillus-Salibacillus Salinicoccus* *Marinococcus*	[11]
Saltern in Huelva (Spain)	lipase protease amylase nuclease	amylase	*Halorubrum* *Haloarcula* *Halobacterium* *Salicola* *Salinibacter* *Pseudomonas*	[15]
Howz Soltan Lake (Iran)	lipase amylase protease xylanase DNase inulinase pectinase cellulase pulullanase	lipase	*Salicola* *Halovibrio* *Halomonas* *Oceanobacillus Thalassobacillus* *Halobacillus* *Virgibacillus* *Gracilibacillus* *Salinicoccus* *Piscibacillus*	[18]
Maharlu Salt Lake (Iran)	protease lipase	ND	*Bacillus* *Paenibacillus* *Halobacterium* *Aeromonas* *Staphylococcus*	[19,20]
Deep-sea sediments of the Southern Okinawa Trough (China)	amylase protease lipase DNase	amylase	*Alcanivorax* *Bacillus* *Cobetia* *Halomonas* *Methylarcula* *Micrococcus* *Myroides* *Paracoccus* *Planococcus* *Pseudomonas* *Psychrobacter* *Sporosarcina* *Sufflavibacter* *Wangia*	[25]
Slanic Prahova salt mine (Romania)	amylase gelatinase lipase protease cellulase xylanase	lipase protease	ND	[27]
Atacama Desert (Chile)	amylase protease lipase DNase xylanase pullulanase	DNase	*Bacillus* *Halobacillus* *Pseudomonas* *Halomonas* *Staphylococcus*	[28]
Saline desert “Indian Wild Ass Sanctuary” (India)	amylase	ND	*Bacillus*	[32]

ND: Not determined

### 2.2. Screening on Salt Lakes

In a hypersaline lake in Iran, Rohban *et al.* [18] investigated the ability of halophilic strains to produce different extracellular hydrolases (lipase, amylase, protease, xylanase, DNase, inulinase, pectinase, cellulase and pulullanase) and a wider distribution of hydrolytic activity was observed among Gram-positive bacteria. Extreme halophiles, less represented in comparison with moderate halophiles, showed higher interest as producers of amylases, lipases, cellulases and pectinases. This study reported *Salicola* as the predominant genus among Gram-negative isolates, in contrast with the study of Sanchez-Porro *et al.* [11], according to which most of Gram-negative isolates belonged to the genus *Halomonas*. Among the Gram-positive hydrolase-producing isolates, representatives of the genera *Virgibacillus* and *Thalassobacillus* were predominant. On the other hand, strains of the genus *Salinivibrio* were not found, probably due to the higher concentration of salt in Howz Soltan sea site in comparison with those saline habitats studied in Spain.

Ghasemi and coworkers [19] performed a study focused to identify moderate halophiles from a hypersaline lake in the southern area of Iran. They isolated 16 strains exhibiting proteolytic activity and in comparison to Gram-negative bacteria, the Gram-positive rods displayed higher proteolytic activities. These data are in agreement with other findings previously reported that revealed that Gram-positive bacteria are the dominant proteolytic isolates in the saline environments. Representatives of the genera *Bacillus*, *Paenibacillus, Halobacterium* and *Aeromonas* were described. One species of *Bacillus* was found as the highest protease producer and it was further studied. Other study, concerning lipolytic activity, investigated the presence of halophilic bacteria producing lipases from a Maharlu salt lake in Iran [20]. All strains obtained in this study were moderate halophiles assigned to the genera *Bacillus* and *Staphylococcus*. 

### 2.3. Screening on Saline Deposits

Deep-sea sediments have also been considered interesting habitats as source of novel enzymes, due to their extreme conditions; however, they have been practically unexplored on earth [21,22,23,24]. For this reason, Dang *et al.* [25] screened deep-sea sediments of the Southern Okinawa Trough in order to show the diversity and abundance of bacterial isolates secreting enzymes (amylases, proteases, lipases, DNases and chitinase). The isolates on this study were quite diverse and a total of 14 different genera were identified, including *Alcanivorax*, *Bacillus*, *Cobetia*, *Halomonas*, *Methylarcula*, *Micrococcus*, *Myroides*, *Paracoccus, Planococcus, Pseudomonas, Psychrobacter, Sporosarcina, Sufflavibacter* and *Wangia.* Bacteria of the *γ-Proteobacteria* lineage, especially those from the *Halomonas* and *Psychrobacter* groups, dominated in the culturable bacteria assemblage. This predominance could indicate that this population is ubiquitous in marine sediments, at least in the west Pacific and the distinct distribution of the other bacterial groups might indicate that their distributions could be restricted by certain environmental conditions.

According to the studies of Sánchez-Porro *et al.* [11] and Moreno *et al.* [15], the strains producing amylolytic enzymes were the most diverse and abundant physiological group among the hydrolytic producers. Some isolated strains even harbored all the extracellular hydrolytic activities screened, except for the chitinase activity. The lack of chitinase activity on the bacterial isolates could indicate that the terrestrial export of the particulate organic matters may be the major source of the biopolymers buried in the studied deep sea sediments. Dell' Anno and Danovaro [26] suggested that the microbial degradation of extracellular DNA in deep-sea ecosystem might provide another suitable C and N source for sediment prokaryote metabolism.

In a similar study, Cojoc and colleagues [27] elucidated the extracellular hydrolytic activities of halophilic bacteria collected from a salt deposit having 45.5 to 499 m deep. The lipolytic and proteolytic activities were predominant among the isolated strains and one of the strains hydrolyzed six different substrates. The relatively low number of microorganisms in the investigated environment can be correlated with the low temperature exhibited by the investigated area. 

### 2.4. Screening on Saline Deserts

Recently, in heavy-metal-contaminated soils with extreme salt conditions from the Atacama Desert, Moreno *et al.* [28] carried out a screening to isolate hydrolytic enzyme producers. The most frequent hydrolytic activity detected in the study was DNase, followed by amylase and lipase in contrast with the studies of Sánchez-Porro *et al.* [11] and Moreno *et al.* [15]. In the analyzed community, pullulanase and protease producers were also detected, being xylanases the least represented among the hydrolases tested. It is interesting to emphasize that multiple hydrolytic activity was frequently detected in the isolates reported in this study supporting previous studies in other hypersaline habitats [11,15]. As reported in the study of Rohban *et al.* [18], most environmental isolates able to produce hydrolytic enzymes were Gram-positive bacteria, although the isolates were assigned to the family *Bacillaceae*, comprising species of the genera *Bacillus, Halobacillus* and *Thalassobacillus*. Only two isolates were related to the Gram-negative bacteria *Pseudomonas halophila* [29] and *Halomonas organivorans* [30] and the other characterized isolates were related to *Salinicoccus roseus* [31].

Khunt *et al.* [32] isolated from an Indian saline desert environment, moderate halophiles able to produce extracellular amylases and they reported that isolates were Gram-positive, non-capsulated bacteria assigned to the genus *Bacillus*. 

In summary, we can conclude that there is a wide taxonomic diversity of microorganisms showing hydrolytic activity in saline environments and in most cases multiple activities are present. Aerial distribution of the dormant spores probably explains the occurrence of *Bacillus* in most habitats. This genus is well known as an extracellular enzyme producer and many industrial processes use species of this genus for commercial production of enzymes [33].

## 3. Biotechnological Potential of Bacterial Halophilic Hydrolases

Although halophilic enzymes are considered a novel alternative for use as biocatalysts in different industries, there are relatively few studies on halophilic enzymes, with some being based on their isolation and others on their characterization. However, it is important to thoroughly study these enzymes in order to use them in biotechnological processes [8,34]. Only a few industrial applications of halophilic enzymes, mainly in the manufacturing of solar salt from seawater, fermented food, textile, pharmaceutical and leather industries, have been reported. Highlighted halophilic intracellular or extracellular hydrolases mentioned in this review are summarized in the Table 2.

### 3.1. Hydrolases Produced by Moderately Halophilic Bacteria

#### 3.1.1. Bacterial Lipolytic Enzymes

Bacterial lipolytic enzymes are valuable biocatalysts due to their broad substrate specificity and high chemo-, regio- and stereoselectivity [35,36,37,38,39,40]. Thus, these enzymes are currently used as detergent additives, in the food and paper industries, and as enantioselective biocatalysts for the production of fine chemicals [37,41,42,43,44,45,46]. However, industrial applications of lipases are often hampered by their low stability in the processes, including low thermostability and loss of activity in presence of the organic solvents, where most of reactions are performed. In this sense, the lipases isolated from extreme microorganisms constitute an excellent alternative in the industrial processes [47].

**Table 2:** Selected enzymes from extremely and moderately halophilic bacteria with potential biotechnological applications.

Source	Bacteria	Enzyme	Localization	References
Extremely halophilic bacteria	*Salicolamarasensis* sp. IC10	Lipase LipL	Extracellular	[15]
		Protease SaliPro	Extracellular	[15]
Moderately halophilic bacteria	*Marinobacter* *lipolyticus*	Lipase LipBL	Intracellular	[48,49,50]
	*Pseudoalteromonas ruthenica*	Haloprotease CP1	Extracellular	[51,54]
	*Halobacillus* *karajensis*	Protease	Extracellular	[55]
	*Nesterenkonia* sp. strain F	α-amylase	Extracellular	[57]
	*Thalassobacillus* sp. LY18	α-amylase	Extracellular	[58]

An interesting intracellular lipolytic enzyme produced by the moderately halophilic bacterium *Marinobacter lipolyticus* SM19 was isolated and characterized [48]. This enzyme, designated LipBL, was assigned to the family VIII of lipolytic enzymes and it was expressed in *Escherichia coli*. LipBL is a protein of 404 amino acids with a molecular mass of 45.3 kDa and high identity to class C β-lactamases. LipBL was purified and biochemically characterized. The temperature for its maximal activity was 80 °C and the pH optimum determined at 25 °C was pH 7.0, showing optimal activity without sodium chloride, while maintaining 20% activity in a wide range of NaCl concentrations. This enzyme exhibited optimal activity against short-medium length acyl chain substrates, although it also hydrolyzes olive oil and fish oil. The enzyme is also active towards different chiral and prochiral esters. Exposure of LipBL to buffer-solvent mixtures showed that the enzyme had remarkable activity and stability in all organic solvents tested. For improving the stability and for use in industrial processes LipBL was immobilized in different supports [48]. 

The fish oil hydrolysis using LipBL results in an enrichment of free eicosapentaenoic acid (EPA), but not docosahexaenoic acid (DHA), relative to its levels present in fish oil. The fish oil used in the experiment contained 18.6% of EPA, increasing to 27% after the treatment with LipBL-CNBr immobilized derivative, representing an increase of 45.2% of the total concentration, however, the content in DHA only increase from 12% to 12.5%, representing an increase of 4.2%. These results indicated that LipBL showed more selectivity towards the hydrolysis of DHA increasing EPA, being a good candidate for use at an industrial scale for the production of fish oils enriched in polyunsaturated fatty acids (PUFAs) [48,49]. Due to its biotechnological interest, LipBL mutants were obtained in order to determine the influence of different residues in the functionality of LipBL. Mutants were constructed by site directed mutagenesis obtaining LipBL variants with different properties (specificity to different substrates, optimal pH values and thermostability) [50].

#### 3.1.2. Bacterial Proteases

Proteases constitute one of the most important groups of industrial enzymes, comprising currently the majority of worldwide enzyme sales. They have been widely used in industry for a long time, especially in washing detergent, baking, brewing, cheese industry and tanning industry [51,52]. Due to the stability and properties of halophilic proteases, these enzymes are good candidates for use in industrial processes. An interesting extracellular protease, designated haloprotease CP1 has been isolated from the moderately halophilic bacterium *Pseudoalteromonas ruthenica* [51]. The maximal production of the protease CP1 by *P. ruthenica* CP76 was detected at the end of the exponential growth phase at 37 °C, in media containing 7.5% salt and supplemented with sucrose (50 mM). Protease CP1 was purified from *P. ruthenica* CP76 using ion exchange and gel filtration chromatography. The purified enzyme was biochemically characterized, showing optimal activity at 55 °C, pH 8.5 and high tolerance to a wide range of NaCl concentrations (0 to 4 M NaCl). It has a molecular mass of 38 kDa and the activity was strongly inhibited by ethylenediamunetetraacetic acid (EDTA), phenylmethylsulfonyl fluoride (PMSF) and Pefabloc [53,54]. Another enzyme characterized was the protease produced by the moderately halophilic bacterium *Halobacillus karajensis* strain MA-2. Effect of various temperatures, initial pH, salt and different nutrient sources on protease production revealed that the maximum secretion of the enzyme occurred at 34 °C, pH 8.0–8.5, and in the presence of gelatin. The maximum enzyme activity was obtained at pH values ranged from 8.0 to 10.0, with 55% and 50% activity remaining at pH 6 and 11, respectively. Moreover, the enzyme activity was strongly inhibited by PMSF, Pefabloc SC and EDTA; indicating that this enzyme probably belongs to the subclass of serine metalloproteases. These findings suggested that the protease secreted by *Halobacillus karajensis* presents a great potential for biotechnological applications when alkaline conditions are required [55].

#### 3.1.3. Bacterial Amylases

Amylases constitute a group of interesting enzymes from the biotechnological point of view [56]. Most interesting applications are in the clinical and analytical chemistries, as well as their widespread applications in starch saccharification and in the textile, food, brewing and distilling industries [56], where halophilic amylases highlighted due to their stability and versatility. In the last years, several extracellular halophilic α-amylases have been purified from moderate halophiles. One of them was purified from *Nesterenkonia* sp. strain F [57]. This enzyme showed an apparent molecular weight of 110 kDa by SDS-PAGE and it exhibited maximal activity at pH 7–7.5, being relatively stable at pH 6.5–7.5. Optimal temperature for the amylase activity and stability was 45 °C. The purified enzyme was highly active in a broad range of NaCl concentrations (0–4 M) with optimal activity at 0.25 M NaCl. This amylase was highly stable in the presence of 3-4 M NaCl and the activity was not influenced by Ca^2+^, Rb^+^, Li^+^, Cs^+^, Mg^2+^ and Hg^2+^, whereas Fe^2+^, Cu^2+^, Zn^2+^ and Al^2+^ strongly inhibited the enzyme. Moreover, this α-amylase was inhibited by EDTA, but was not inhibited by PMSF and β-mercaptoethanol [57]. 

An extracellular halophilic α-amylase was purified from *Thalassobacillus* sp. LY18 [58]. This enzyme showed a molecular mass of 31 kDa and its optimal enzyme activity was found to be at 70 °C, pH 9.0, and 10% NaCl. The α-amylase was highly stable over a broad range of temperatures (30–90 °C), pH (6.0–12.0), and NaCl concentrations (0%–20%), showing excellent thermostable, alkalistable, and halotolerant nature. The enzyme was stimulated by Ca^2+^, but greatly inhibited by EDTA, indicating it was a metalloenzyme. Complete inhibition by diethyl pyrocarbonate and β-mercaptoethanol revealed that histidine residue and disulfide bond were essential for enzyme catalysis. Furthermore, it showed high activity and stability in the presence of water-insoluble organic solvents with log P (ow) ≥ 2.13 [58].

### 3.2. Hydrolases Produced by Extremely Halophilic Bacteria. Bacterial Lipolytic and Proteolytic Enzymes

On the other hand, there are some hydrolases characterized from extremely halophilic bacteria. The extremely halophilic bacterium *Salicola marasensis* IC10 produces an extracellular protease, designated Salipro, and at least an intracellular lipase, which was named LipL [15]. The lipolytic activity of the *S. marasensis* IC10 is mainly located in the cytoplasmatic fraction. This enzyme is active in presence of different compounds as substrates: *p*-nitrophenyl butyrate, *p*-nitrophenylvalerate, *p*-nitrophenylcaprilate and *p*-nitrophenyldecanoate as well as 4-methylumbelliferone and the enzyme production is maximal at the end of the exponential phase. The effect of different factors on the lipolytic activity was analyzed and the maximum values were obtained in the presence of 1 M NaCl, although the tolerance was found in the range from 0 to 4 M NaCl, with 6 mM of betaine. The lipolytic activity increased in the presence of organic solvents as 1-buthanol, 2-buthanol and acetone (5% and 10%, v/v), EDTA 1% (v/v) and metal ions as Ni^2+^ and Ca^2+^.

To identify the optimal conditions for protease production, the characterization of the intracellular fraction of *Salicola* sp. IC10 during growth was performed, finding the optimal conditions at pH 8.0, 40 °C and a medium with 15%–20% (w/v) NaCl. Thus, there is a correlation between the optimal conditions for cultivation of the strain and the maximum production of the proteolytic enzyme. This protease showed the capability to effectively catalyze the hydrolysis of various proteins. The most specific substrate to the enzyme was egg albumin, followed by gelatine (97% relative activity) [15]. 

Therefore, we can conclude that enzymes produced by halophilic bacteria show interesting properties for use in different industries.
